# Supplementary material for: The Small RNA Universe of Capitella teleta
Source: Front Mol Biosci. 2022 Feb 25;9:802814. doi: 10.3389/fmolb.2022.802814 (PMC8915122; doi:10.3389/fmolb.2022.802814)
Supplement: Supplementary file 1 [file DataSheet1.ZIP › Supplement/known/CAPTEscaffold_876_29814.pdf]

Provisional ID	:	CAPTEscaffold_876_29814
Score total	:	1837.5
Score for star read(s)	:	3.9
Score for read counts	:	1828.4
Score for mfe	:	0.6
Score for randfold	:	1.6
Score for cons. seed	:	3
Total read count	:	3598
Mature read count	:	3465
Loop read count	:	0
Star read count	:	133

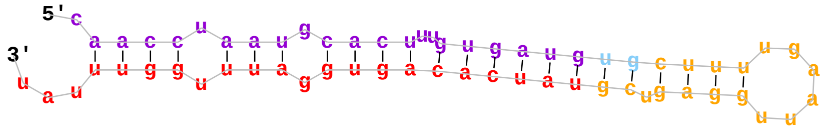

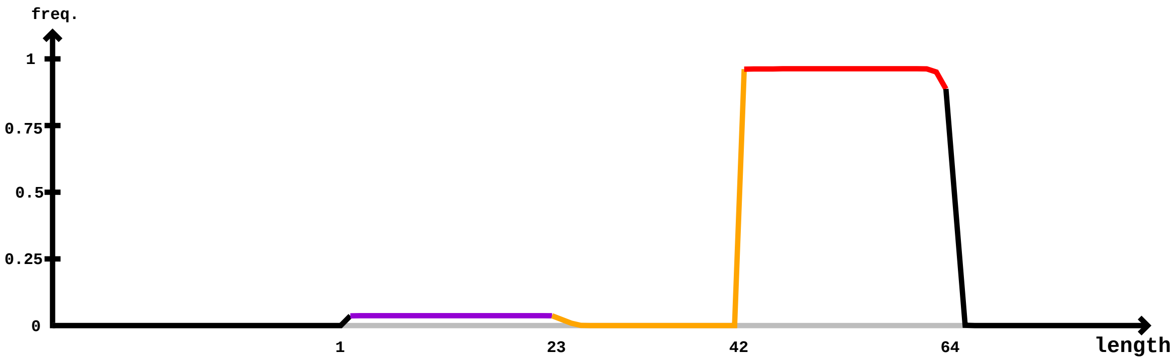

Star Mature

```
5'- cucuuggcuuuuucaagcuuuugagaagucaaccuaaugcacuuugugaugugcuuuugaauuggagucguaucacaguggauuugguuuauuucuuacauuuucuaucauc   -3'   obs
    cucuuggcuuuuucaagcuuuugagaagucaaccuaaugcacuuugugaugugcuuuugaauuggagucguaucacaguggauuugguuuauuucuuacauuuucuaucauc         exp
    cucuuggcuuuuucaagcuuuugagaagucaaccuaaugcacuuugugaugugcuuuugaauuggagucguaucacaguggauuugguuuauuucuuacauuuucuaucauc         known
    .....(((((....)))))..((((((((.((((.(((.((((..((((((((((((......)))).))))))))))))).))).)))).))))))))..............   reads   mm   sample

    .............................caaccuaaugcacuuugugau..............................................................   1       0    seq
    .............................caaccuaaugcacuuugugaug.............................................................   59      0    seq
    .............................caaccuaaugAacuuugugaug.............................................................   1       1    seq
    .............................caaccuaaugcacuuugugauA.............................................................   1       1    seq
    .............................caaccuaaugUacuuugugaugu............................................................   2       1    seq
    .............................caaccuaaugcacuuugugUugu............................................................   1       1    seq
    .............................Uaaccuaaugcacuuugugaugu............................................................   1       1    seq
    .............................caaccuaaugcacuuugugaugu............................................................   35      0    seq
    .............................caaccuaaugcacuuugugaugug...........................................................   28      0    seq
    .............................caaccuaaugcacuuugugaugugU..........................................................   2       1    seq
    ..............................aaccuaaugcacuuugugaugug...........................................................   2       0    seq
    ......................................................................uaucacaguggauuugguu.......................   2       0    seq
    ......................................................................uaucacaguggauuugguuu......................   41      0    seq
    ......................................................................uaucacaguggaGuugguuu......................   1       1    seq
    ......................................................................uaucacaguggauuGgguuua.....................   1       1    seq
    ......................................................................uaucacaguggauuugguuua.....................   227     0    seq
    ......................................................................uaucacaguggauuugguuuU.....................   1       1    seq
    ......................................................................uauAacaguggauuugguuuau....................   1       1    seq
    ......................................................................uaucacaguggauuugguuuaC....................   6       1    seq
    ......................................................................uaucacaguAgauuugguuuau....................   14      1    seq
    ......................................................................uaucacaguggauuuAguuuau....................   3       1    seq
    ......................................................................uaucacagGggauuugguuuau....................   1       1    seq
    ......................................................................uaucacaguggauuugguAuau....................   1       1    seq
    ......................................................................uaucUcaguggauuugguuuau....................   1       1    seq
    ......................................................................uaucacUguggauuugguuuau....................   2       1    seq
    ......................................................................uaucacaguggauuCgguuuau....................   2       1    seq
    ......................................................................uaucacaguggauuugCuuuau....................   1       1    seq
    ......................................................................uauUacaguggauuugguuuau....................   2       1    seq
    ......................................................................uaucacaguggaGuugguuuau....................   1       1    seq
    ......................................................................uaucacagugAauuugguuuau....................   2       1    seq
    ......................................................................uaucacaguggauuuUguuuau....................   1       1    seq
```

```
                              Star                                      Mature
cucuuggcuuuuucaagcuuuugagaagucaaccuaaugcacuuugugaugugcuuuugaauuggagucguaucacaguggauuugguuuauuucuuacauuuucuaucauc
......................................................................uaucacaguggauuuggCuuau....................    1       1       seq
......................................................................uaucacagAggauuugguuuau....................    4       1       seq
......................................................................uaucacaguggauuugguuuau....................    2961    0       seq
......................................................................uaucacaguggauAugguuuau....................    2       1       seq
......................................................................Aaucacaguggauuugguuuau....................    10      1       seq
......................................................................uaucacaguggauuugguuuaA....................    3       1       seq
......................................................................uaucacaguggauuugguuuGu....................    2       1       seq
......................................................................uaucacaguCgauuugguuuau....................    1       1       seq
......................................................................uaucacaguggauuAgguuuau....................    1       1       seq
......................................................................uaAcacaguggauuugguuuau....................    8       1       seq
......................................................................Gaucacaguggauuugguuuau....................    1       1       seq
......................................................................uaucacaguggaAuugguuuau....................    1       1       seq
......................................................................uGucacaguggauuugguuuau....................    2       1       seq
......................................................................uaucaAaguggauuugguuuau....................    1       1       seq
......................................................................uaCcacaguggauuugguuuau....................    2       1       seq
......................................................................Aaucacaguggauuugguuuauu...................    2       1       seq
......................................................................uaucacaguggauuugguuuauu...................    99      0       seq
......................................................................uaucacaguAgauuugguuuauu...................    1       1       seq
......................................................................uaucUcaguggauuugguuuauu...................    1       1       seq
......................................................................uaucacaguggauuugguuuauG...................    4       1       seq
......................................................................uaucacaguggauuugguuuauA...................    32      1       seq
......................................................................uaucacaguggauuugguuuauC...................    5       1       seq
......................................................................uaucacaguggauuugguuuauuu..................    3       0       seq
......................................................................uaucacaguggauuugguuuauCu..................    1       1       seq
.......................................................................aucacaguggauuugguuuau....................    3       0       seq
..........................................................................acaguggauuugguuuau....................    3       0       seq
```
